# Supplementary material for: Are carbon emissions trading and green financial instruments synergistic? -Comprehensive quantitative research based on content analysis
Source: PLoS One. 2024 Mar 7;19(3):e0298601. doi: 10.1371/journal.pone.0298601 (PMC10919601; doi:10.1371/journal.pone.0298601)
Supplement: S1 Data — (ZIP) [file pone.0298601.s001.zip › Data/地级市Z_F工作报告.zip!/╝¬┴╓/╝¬┴╓╩╨/└┤╫╘╣1⁄2╓┌║┼í╛┬φ┐╦╩2╛▌═°í┐_2015.docx]

各位代表：

　　现在，我代表市政府向大会报告工作，请予审议，并请市政协委员提出意见。

　　一、2014年工作回顾

　　去年，面对宏观环境错综复杂、经济下行压力加大的严峻形势，我们在省委、省政府和市委的正确领导下，在市人大、市政协的大力支持下，统筹稳增长、调结构、促改革、惠民生，扎实进取，奋力拼搏，经济社会保持了稳中有进、稳中向好的态势。预计实现地区生产总值2753亿元，增长6.1%；固定资产投资2300亿元，增长15%；社会消费品零售总额1195亿元，增长12%；地方级财政收入131.2亿元，低于上年水平，其中市本级增长6.7%；城镇居民人均可支配收入28530元，农村居民人均纯收入11530元，分别增长10%和12%。

　　——突出抓经济运行，稳增长取得新成效

　　把稳工业作为稳增长的关键，采取挖潜增效、减亏增盈、银企保对接、产需衔接等一系列针对性措施，工业经济在严重困难的情况下逐步企稳，全市规模工业增加值、利润分别增长5.3%和15%。突出功能区领先发展，十大功能区实现GDP、地方级财政收入、规模工业增加值占全市比重分别达到29%、24%和45%。大力实施民营经济“双50、双100”工程，深入推进全民创业，新增“四上”企业300户，新生成民营企业4900户，民营经济对地方级税收贡献率达到73.5%。地产品与大型商超对接、“十城百店千品进万家”等活动取得明显成果，欧亚商都综合体、红星美凯龙家居生活广场等一批重点商贸项目投入运营，东北首家区域性电子商务平台——统泰“一网全城”上线运营。加强对外营销推介，雾凇冰雪节、松花江河灯节等品牌节庆活动影响持续扩大，旅游总收入增长25.5%。抢抓新一轮振兴东北机遇，哈达湾老工业区搬迁改造等100个项目纳入国家支持范畴，丰满大坝重建、万丰工业园等一批重点项目进展顺利，30万吨环氧丙烷产业链等348个项目竣工投产，全年实施亿元以上项目564项、10亿元以上54项。务实开展招商活动，万达城市广场、海王健康产业生产制造基地等359个招商项目开工建设。

　　——坚持抓转型升级，调结构迈出新步伐

　　产业结构调整进程加快。累计投入技改资金639亿元，吉化柴油质量升级、晨鸣纸业异地迁建等一批重大技改项目建成投产，化工、汽车、冶金等传统产业精深加工程度进一步提升。战略性新兴产业发展态势良好，恒辉10万吨高性能改性材料等一批重点项目竣工运行，150种重点新产品实现规模化生产。高起点规划建设医药健康和通用航空产业，医药健康产业产值增长27.6%，支线机场综合配套改革试点申报工作进展顺利。保税物流中心获批建设。实施市级以上重大科技成果转化和重点科技攻关项目141项，科技成果转化率达到50%。博宇医药物流园、安中危化品物流服务中心投入运营。大仝、中讯等软件企业发展迅速，软件外包业收入增幅超过40%。银行业金融机构发展到15家，存贷款余额保持10%以上增速。粮食总产量稳定在110亿斤阶段水平，特产业产值增长16%，畜禽规模养殖比重达到86%，无规定疫病区建设走在全省前列。“万昌”、“天德盛”等获得国家地理标志产品保护和地理标识注册商标，“大荒地大米”等商标被认定为中国驰名商标，“中国粳稻贡米之乡”品牌效应扩大。空间结构布局优化。中心城市综合服务功能和辐射带动作用有效增强，实施火车站西广场、人民大剧院等158项城建重点工程，雾凇大桥、吉林大桥等8座桥梁建成通车，雾凇西路等28条街路竣工，长吉北线全线贯通，污水处理厂二期、五水厂提标改造工程投入运行。客运市场、违章建筑等专项整治行动收到实效，城市管理效率和质量进一步提升。加强县城基础设施建设，完善公共服务功能，各具特色的县城发展新形象进一步展现。6个省市示范镇建设稳步推进。

　　——统筹抓重点领域改革，促改革实现新突破

　　经济体制改革成效明显。实施工商注册登记制度改革，新登记企业、注册资本分别增长47%和116%。组建市国有资本投资运营公司，建立市属国有企业董事会、监事会向市政府报告工作制度，初步实现由“管资产”向“管资本”的有效转变。金融控股集团成立，创新“种子基金”、“助保金池”等金融产品，金洪汽配在“新三板”挂牌，吉林化纤、金浦钛业等企业定向增发股票募集资金95亿元。新增家庭农场410个、农民专业合作社1200个，土地流转面积8.3万公顷，

完成30个试点村土地确权登记颁证工作。行政体制改革不断深化。推进简政放权，减少行政审批项目226项，精简幅度35%；实施重点项目并联审批流程再造，压缩审批时限60%以上。市县两级卫计委、食药监、工商、质监管理体制调整基本到位，事业单位分类工作基本完成。财政预算、部门预算向社会公开。社会领域改革同步推进。全面实行“阳光招生”、“阳光入学”、“阳光分班”，普惠、优质教育取得明显成果；省内最大中等职教园区启动建设。城区职工医保实现“同城同待遇”，城乡居民参保基本实现全覆盖；基本药物零加价销售，累计为群众让利1.33亿元。

　　——全力抓民生改善，惠民生展现新成果

　　总投入110亿元的77项民生实事全面完成。城镇新增就业9.5万人，农村劳动力转移72万人次。城市、农村低保标准分别上调到每人每月388元和每人每年2572元。全市企业退休（退职）人员养老金实现“十连调”，月人均养老金平均增幅10%。改造棚户区109万平方米，建设廉租房、公租房2035套、11万平方米，1.25万户低收入家庭享受到廉租住房租赁补贴，完成“暖房子”工程改造230万平方米。新建、改造温室大棚2000栋，新增蔬菜生产能力5000万斤。建成平价直销便利店152家。新增养老床位2000张。开通、调整公交线路58条，新购置公交车350辆。又解决15.2万农村人口饮水安全问题。市中心医院一期、人民医院二期工程投入使用。吉剧《焦裕禄》在国家大剧院公演，广播剧《牛子厚与喜连成》获国家级金奖，成功申办第24届中国金鸡百花电影节，索契冬奥会我市实现竞技体育奥运金牌零的突破。把生态环境建设作为最重要的民生工程，全面开展环境综合整治。淘汰落后产能57万吨，集中供热率达到全省最高的95%，城市饮用水源地水质达标率100%，主城区噪音指标控制在55分贝以内，人均拥有公共绿地面积超过12平方米。清收还林成效显著，清收林地121万亩、造林75万亩，分别完成年度任务的146%和150%。安全稳定的社会局面得到持续巩固。

　　把开展党的群众路线教育实践活动作为重大政治任务，以“钉钉子”精神集中解决“四风”突出问题，会议、文件量分别削减57.4%和50%，“三公”经费同比下降42.9%，清理超标公车707辆，腾退办公用房9.5万平方米，建立健全规章制度428项，一大批群众反映强烈的难点问题得到有效解决，政府系统服务水平、工作效能明显改进，加强作风建设的常态化、长效化机制初步形成。

　　各位代表，过去一年，吉林市能够在逆境中砥砺前行，在困难中有效突破，得益于我们一手抓教育实践活动开展，一手牢牢把发展作为第一要务，做到了两手抓、两促进；得益于我们立足发展实际，强化“稳”的针对性举措，挖掘“增”的优势潜力，用改革创新的办法，开放“上争”的力度，夯实了老工业基地调整转型的基础；得益于我们始终坚持科学施政、依法行政，不断增强制度约束力、行政执行力和工作落实力，以求真务实的作风、团结协作的精神，铸就了来之不易的发展成果。在此，我代表市政府，向全市各族人民，向所有参与、关心和支持吉林市发展的同志们、朋友们，表示衷心的感谢和崇高的敬意！

　　我们也看到，全市发展中仍然存在不少问题与不足：经济企稳向好的基础还不牢固，部分骨干企业生产经营困难，项目建设步伐不快，财政增收难度加大，发展内生动力不足。调结构任重道远，基础原材料产业比重偏大、民营经济不发达、现代服务业贡献率低的状况还没从根本上得到改变。公共服务能力有待进一步提升，交通出行、教育医疗、生态环境等与广大群众的新期盼相比还有一定差距。转变政府职能还需花更大气力，有的部门和个别干部服务意识不强、效率不高，抓落实不到位的现象依然存在。对此，我们一定找准症结，统筹施策，认真加以破解。

　　二、2015年工作安排

　　2015年，是我市改革发展进程中十分关键的一年，新常态成为主要特征。尽管现阶段宏观形势依然复杂严峻，但国家新一轮振兴东北和长吉图开发开放战略的实施，以及全省中部创新转型核心区和长吉产业创新发展示范区建设的大力推进，为我们提供了千载难逢的重大机遇。我们一定要充分认识到吉林市在全省发展大局中的核心地位和肩负率先发展的职责使命，进一步树立强烈的大局意识、责任意识和担当精神，集中发力，克难攻坚，力争为全省发展做出更大贡献。

　　政府工作总体要求是：深入贯彻党的十八大、十八届三中四中全会、省委十届四次全会和市委十二届九次全会精神，主动适应经济发展新常态，坚持稳中求进工作总基调，按照突出发挥“五个优势”、推进“五项举措”、加快“五大发展”的要求，把新一轮振兴发展作为总统领，着力推动改革创新，着力加快调整转型，着力保障改善民生，实事求是，奋力作为，努力促进经济平稳健康发展和社会和谐稳定。

　　主要预期目标是：地区生产总值增长7%，规模工业增加值增长6%，固定资产投资增长12%，社会消费品零售总额增长12%，地方级财政收入增长7%左右，城镇居民人均可支配收入、农村居民人均纯收入均增长10%，节能减排完成省下达任务。

　　（一）把稳增长、调结构作为振兴发展的主要支撑，切实提升经济发展质量效益

　　把握稳增长与调结构的平衡点，坚持以增量带动结构优化，以创新促进产业升级，努力推动经济减速不减势、量增质更优。

　　升级改造传统产业。化工行业，着力延伸乙烯、丙烯、碳四碳五等产业链，重点抓好1.5万吨异戊烯醇、4万吨乙丙橡胶等项目建设，提高产品终端化、链式化水平。汽车行业，支持重点企业跨区域战略联合重组、协作配套，吸引整车及零部件企业入驻，加快构建汽车全产业链生产配套体系。冶金行业，大力发展“围钢经济”，抓好恒联铁水连铸铸造基地建设，推进中钢吉铁、中钢吉炭实施重组和搬迁改造，做精做深钢铁产业链条。

　　培育壮大战略性新兴产业。突出重大项目建设、领军企业培育、关键技术突破等环节，加快发展新材料、生物技术、信息技术、医药健康和通用航空产业，组织实施100项战略性新兴产业重点项目，抓好100种新产品规模化生产，全市战略性新兴产业产值增长10%以上。

　　融合发展服务业。坚持现代服务业与传统服务业相融合。完善化工、汽车等专业物流园区，建设东北亚综合物流园、亚琦国际物流中心等物流基地，壮大第三方物流产业；培育发展科技服务业、总部经济、楼宇经济和健康养老等新的消费增长点。改造提升老商圈，积极发展哈达湾、越北、临江门、南部新城等区域商业中心，加快万达城市广场、吉林国际商贸城等重点项目建设，新增限上企业50户。坚持文化产业与旅游产业相融合。注重挖掘文化内涵，保护性开发松花湖、北大壶、朱雀山等旅游景点，推出一批独具文化特色的精品旅游产品，旅游总收入增长25%以上。

　　推进农业发展方式转变。抓好水稻高光效推广技术应用，农作物耕种收综合机械化水平达到73%，粮食生产保持110亿斤阶段水平。推动粳稻贡米产业向标准化生产、产业化经营、精深化加工发展，提高“吉林市大米”品牌效应和市场竞争力。积极发展休闲农业，新增省级以上星级企业5户，休闲农业经营主体发展到280个。抓好永吉国家级农业示范区建设。发展壮大甜粘玉米、紫苏、芦笋等特产业，推进种植业结构调整。新建改建210个养殖小区，完成7项无规定疫病区建设任务。加快大润德等综合开发项目建设，新增市级以上龙头企业15户。实施农田水利基础设施建设313项。

　　坚持投资拉动。实施3000万元以上重点项目900项，其中亿元以上项目550项；竣工3000万元以上项目350项；产业链延伸、战略性新兴产业和现代服务业项目占全部投资比重提高到60%。分类梳理近年来重点建成项目，落实帮扶措施，达产达效率达到80%以上。储备亿元以上项目300项，项目实施率超过30%。有效利用全国工商联、各地商会等平台，有针对性地开展点对点招商、专业招商、以商招商等活动。全年新开工3000万元以上招商项目400项，实现招商引资到位资金1050亿元。

　　（二）把创新驱动作为振兴发展的有效途径，切实增强内生发展动力

　　围绕创建国家创新型试点城市，挖掘创新资源，集聚创新要素，不断增强核心竞争力和可持续发展能力。

　　推进科技创新。深入实施产业创新示范工程，扶持精细化工等9个产业集群发展壮大。启动科技“小巨人”企业培育计划，抓好科技企业孵化器、技术创新中心和创新团队建设，切实提升企业创新能力。建立区域创新平台，推动吉林大学工业技术研究院和东北电力大学、化工园区科技园建设，深化与中科院、北京化工大学等合作，组建产业技术创新战略联盟。实施100个市级以上重大科技成果转化和重点科技攻关项目，推进30项科技成果转化为实际生产力，提高科技成果产业化水平。

　　加快金融创新。完成华夏银行吉林支行组建，每个县（市）区、开发区发展1家村镇银行。推进3-5户企业上市融资；通过发行企业债、公司债、中期票据等方式多元融资，提高资产证券化水平。拓展“种子基金”、“助保金池”等业务功能，推广产业夹层基金业务，探索开展物权融资模式。启动实施国家"PPP"示范项目，设立产业引导投资基金，带动社会资本创业投资。壮大都邦保险公司规模，提升金融控股集团融资能力。加大支持力度，加快融资担保行业发展。加强信用体系建设，努力改善金融生态环境。

　　积极发展互联网经济。以建设国家电子商务示范城市为重点，推进互联网与传统产业、新兴产业融合发展。抓好高新区电子商务总部基地、大仝软件服务外包基地等重点项目建设，完善“一网全城”电子商务平台功能，扶持吉福参等10家电商企业快速发展。大力推进电子商务进农村，集中打造一批电商村镇和“村淘”试点。加快云计算应用示范推广，推动中油吉林数据中心、江南数据中心商业化运营。提升中小企业信息化应用水平，创建“数字企业”100户、电子商务应用示范企业100户。以整合资源、信息惠民为核心，规划建设智慧城市。

　　实施人才和质量强市战略。坚持培养引进激励并举，壮大杰出青年人才和科技创新创业领军人才队伍，鼓励高校、科研院所科技人员为企业服务和自主创业；加快高新北区职教园区建设，完善多样化职业技能人才培养模式；认真落实人才政策，健全人才评价选拔体系，营造尊重人才、推崇创造、全民创新的社会环境。引导企业牢固树立“质量第一”的经营理念，扶持企业争创省政府质量奖、市长质量奖和名牌产品，提升企业质量管理制度创新和产品创新水平。

　　（三）把深化改革作为振兴发展的核心引领，切实激发经济社会发展活力

　　用好政府放权让利的“减法”，充分调动社会创新创造热情的“乘法”，有效发挥市场在资源配置中的决定作用和更好地发挥政府作用。

　　1.推进行政审批制度改革。深化简政放权，进一步加大市级事权清理力度，再下放一批审批权限，做到放得下、接得住、管得好。建立统一规范的公共资源交易体制机制，加快市公共资源交易中心建设。

　　2.推进机构改革。以提高服务水平倒逼政府改革，推进政府组织机构、职能配置、运行方式法治化。减少部门职责交叉和分散，做好不动产登记、城乡居民基本医疗保险职责整合等相关工作。深化事业单位分类改革。

　　3.推进国资国企改革。完善国有企业出资人制度，从国资监管上实现企业全覆盖、事项全覆盖。推行外派监事会和国有产（股）权代表制度。在法人治理结构、业绩考核、薪酬管理等方面，对国有企业实行分类管理。明确企业功能和主业，推动国有资本逐步从不具备竞争优势的领域和业务中有序退出。

　　4.推进市场体系改革。深化工商注册登记制度改革，开展企业准入“单一窗口”试点；落实支持民营经济发展的各项政策措施，全面营造促进民营经济发展的社会氛围，民营经济主营业务收入增长10%以上。大力培育技术服务企业，逐步放开建筑领域技术服务市场。

　　5.推进财税体制改革。认真贯彻预算法，完善政府预算体系，实行全口径预算管理。健全以项目为导向的政府购买公共服务机制。强化政府性债务管理，完善债务风险预警及化解机制。

　　6.推进教育医疗体制改革。做好适应高考制度改革相关工作，实行城区义务教育“大学区”制，理顺义务教育阶段学校管理体制，支持民办教育发展。完善城市医疗资源布局，支持社会力量创办医疗机构。

　　7.推进农村综合改革。新增家庭农场和农民专业合作社850个，流转土地达到11万公顷，完成28个乡镇452个村土地确权登记颁证任务。推广土地收益保证贷款试点经验，启动林权交易金融服务中心建设。实施农村集体产权制度改革，探索开展增加农民财产性收入试点工作。稳妥推进供销社和国有林场改革。

　　（四）把建设长吉产业创新发展示范区作为振兴发展的重要抓手，切实加快长吉一体化进程

　　规划设立长吉产业创新发展示范区是省委、省政府作出的重大战略决策。我们要立足发挥自身优势，科学规划布局，明晰发展定位，加强衔接协调，着力开拓长吉两市联动发展的新局面。

　　突出十大功能区建设。统筹推进西北部4个工业功能区合理布局、互动发展；促进中南部4个服务业功能区共建共享、协同融合。强化开发开放，提升中新食品区合作层次，力争晋升为国家级开发区，发挥中新合资开发管理公司作用，推进正大生猪养殖等一批大项目落地建设；推动化工园区与德国巴斯夫、美国杜邦等国际500强企业开展产业链延伸合作。加快项目进区，实施基础设施项目60项，重点产业项目270项。

　　推动两市优势产业融合。借助长春汽车产业的辐射带动效应，发挥我市化工产业优势，大力发展汽车专用料和橡塑零部件产业，重点推进恒辉10万吨高性能改性材料、吉星1500万条子午线轮胎等项目达产达效。支持两市装备制造业间互采互用，扩大我市高低压开关柜、绝缘子等优势产品为长春轨道客车的配套份额。依托建龙300万吨精品钢、80万吨冷轧等规模优势，加快汽车和轨道客车等车用特种钢项目建设步伐。

　　加强区域基础设施和平台建设。围绕两市对进式发展，实施长吉高速6车道、长吉南线改造、双吉火车站等一批重大交通基础设施工程。高标准建设保税物流中心，力争年底前封关运营，构建与长春兴隆综合保税区两翼联动发展格局。立足与长春龙嘉机场错位发展，启动航空产业园建设，推进二台子机场复航和改扩建，抓好中航工业、北京通用航空公司等通航项目建设，全面提高对外联通水平。

　　（五）把新型城镇化作为振兴发展的强大引擎，切实推动城乡统筹发展

　　开展国家新型城镇化试点，努力构建具有我市特色的新型城镇化格局。

　　突出城市开发建设和管理。完善规划体系，推进“多规合一”。新建续建白山大桥等城建重点工程188项，完成投资163亿元。围绕保障性住房建设，强化土地收储功能，实行政府主导集中安置。围绕基础设施建设，新建六水厂，加快二次供水改造，提标污水厂，铺设供热、供水、天然气管道460公里。推进城市管理从繁华市区向居民社区倾斜，从主街路向小街小巷延伸，从低水平向高标准拓展，持续加大对占道经营、违停违建、散流体运输等专项整治力度，提升景观亮化、城市绿化、环境美化、卫生净化水准。以弃管小区为重点，加强物业管理，规范服务标准，提升服务质量。开展“温暖家庭、整洁楼道、和谐社区、畅通街路”建设活动，争创全国文明城市。

　　推进小城镇和县城建设。以产业支撑为核心，抓好金珠、孤店子等4个省级试点和一批市级试点建设，探索解决多元投资、宅基地流转、人口城镇化等问题，打造各具特色的产城融合发展新模式。拓展县城发展空间，组织实施磐石北部核心区、舒兰东部新城区建设等一批重大工程，完备供热、供水、物业管理等公共服务功能，加快五县（市）人口和产业聚集步伐。

　　（六）把保障和改善民生作为振兴发展的最终目的，切实提高城乡居民生活质量水平

　　组织实施十个方面80件民生事项：

　　1.就业增收方面。落实创业就业扶持政策，发放小额担保贷款1.2亿元，扶持创业者2200人；组织农村劳动力、城镇失业人员、残疾人技能培训2.6万人。城镇新增就业9万人，农村劳动力转移65万人次。

　　2.安居工程方面。棚户区和危旧房改造开工120万平方米，建设廉租房、公租房2075套、9.6万平方米，为9000户低收入住房保障家庭发放租赁补贴。实施“暖房子”工程改造140万平方米。投放17亿元公积金贷款解决7000户职工家庭住房难题。加快居民住房产权办理工作。

　　3.医疗保障方面。5所县（市）级医院全面开展公立医院改革并实施基本药物零加价销售；1384个行政村卫生室全部完成标准化建设。城镇职工医保年度内统筹基金、大额补充医疗保险最高支付限额分别提高到9万元和21万元；城镇居民年度内住院医疗费最高支付限额提高到16万元，建立城镇居民大病保险制度，大病保险最高报销额度为30万元。新农合大病保险报销限额达到30万元。

　　4.困难救助方面。建立精准扶贫机制，实现1.5万农村人口脱贫致富。落实社会救助和保障标准与物价上涨挂钩联动机制，城乡低保标准分别达到上年度城镇居民人均可支配收入的20%和农村居民人均纯收入的25%。

　　5.教育助学方面。继续实施教育“阳光行动”，进一步完善“阳光招生”、“阳光入学”、“阳光分班”政策措施。科学配置教育资源，解决江南等区域学校布局不合理问题。实施全市中小学校宽带网络“校校通”、多媒体“班班通”和“平安校园”工程。加强学生德育教育，开展“阳光体育”活动，促进学生素质全面发展。

　　6.文体生活方面。深入开展公益电影放映、童话剧免费公演等系列文化活动，新建、改造城乡居民健身路径设施100处，每个县（市）建设2个残疾人文化体育示范点。启动广电中心建设，抓好满族博物馆、乌拉街“三府”修缮工作。承办好第24届中国金鸡百花电影节。

　　7.交通出行方面。推进东西部快速路、高新大路等道路建设，改造四川街等16条街路，维修滨江南路等街路50万平方米、小街小巷110条。建设3座跨街人行天桥。加强交通组织，优化路网结构和停车管理；规范出租车经营行为；新建公交候车亭100处，开通、整合公交线路27条，再投放300辆公交车。新建、改建农村水泥路200公里。

　　8.生态环境方面。集中并网80%现有燃煤分散供热锅炉，改燃10吨以下商业燃煤锅炉，建成区大型餐饮服务场所全部安装油烟净化装置。综合治理农作物秸秆焚烧。建设10个特色花卉园，改造提升玄天岭、人民广场等10个公园广场，完成20处街头绿化美化，启动20个老旧小区绿化美化社区示范项目。以抓好农村环境清洁等“六大工程”为重点，每个县（市）区确定2个乡（镇）试点先行，打造一批特色美丽乡村。继续打好清收还林攻坚战，清收林地33万亩、造林37万亩。

　　9.公共服务方面。发挥公办福利机构养老托底作用，鼓励社会力量兴办养老机构，新增养老服务床位2200张以上。再解决11万农村居民饮水安全问题。推进肉类蔬菜流通追溯体系试点工程建设，农产品质量安全检测中心投入运行。新建、改造温室大棚400栋，新增冬季蔬菜生产能力380万斤。

　　10.平安创建方面。建立安全生产责任制度和保险管控机制，持续开展安全生产隐患大排查大整改，坚决防止重特大安全事故发生。加强法律援助，引导群众依法表达诉求，妥善解决一批重点信访问题。深入推进“平安吉林”建设，全力维护社会和谐稳定。

　　统筹做好其他重点工作。科学谋划和编制好“十三五”发展规划。认真落实优抚安置政策，创新开展双拥共建活动，争取全国双拥模范城“八连冠”。做好气象、地震、人防、外事、档案等工作。

　　三、建设法治政府

　　深入贯彻落实《中共中央关于全面推进依法治国若干重大问题的决定》，全面推进依法行政，加快建设职能科学、权责法定、执法严明、公开公正、廉洁高效、守法诚信的法治政府。

　　（一）依法全面履行职能。坚持把全民普法和守法作为依法治市的长期基础性工作，深入开展法治宣传教育，努力使尊法守法成为全社会的共同追求和自觉行动。强化政府工作人员法治观念和意识，带头学法、懂法、讲法、守法，提高政府机关运用法治思维和法治方式深化改革、推动发展、化解矛盾、维护稳定的能力。推进政府事权规范化、法律化，制定权力清单，严格按照清单履职用权，做到法无授权不可为；建立责任清单，厘清部门职责边界，明确岗位责任，做到法定职责必须为；推行负面清单，建立行政审批市场禁入标准，做到法无禁止皆可为。

　　（二）健全依法决策机制。把公众参与、专家论证、风险评估、合法性审查和集体讨论决定作为重大决策的法定程序，坚决防止违法决策、“拍脑门”决策现象发生。完善政府法律顾问制度，充分发挥以政府法制机构人员为主体、吸收专家和律师参加的法律顾问队伍作用。推行重大决策终身责任追究制度及责任倒查机制，避免决策失误，提高决策质量。做好物业管理等政府立法工作，做到行政行为于法有据、决策和立法紧密衔接。

　　（三）严格规范公正文明执法。整合行政执法资源，合理配置执法力量，推进城市建设管理、公共卫生、安全生产等领域综合执法，着力解决权责交叉、多头执法问题。加强行政执法队伍建设，狠抓执法纪律和职业道德教育，全面提高执法人员素质。建立健全行政裁量权基准制度，有效避免执法随意性。全面落实行政执法责任制，研究制定重大执法决定审核制度和执法全过程记录制度，解决有法不依、执法不严、违法不究问题。

　　（四）加强行政权力制约监督。全面推进“五权”工作，努力形成科学有效的权力运行制约和监督体系。坚持公开为常态、不公开为例外，全面扩大政务公开和政府信息公开范围，让权力在阳光下运行。认真落实重大事项向市委请示、向人大报告和政协通报制度，自觉接受人大法律监督、工作监督和政协民主监督，高度重视司法监督、舆论监督和社会监督，使政府各项工作在依法行政的轨道上有效运行。

　　各位代表，新时期，党中央从发展战略全局的高度，提出了一系列治国理政的新思路、新任务和新要求，我们一定切实增强“六种意识”、提高“六种能力”，巩固教育实践活动成果，努力在新常态中实现新作为。

　　进一步加强政府系统党风廉政建设，廉洁从政、干净干事，以从严的态度、从严的标准、从严的举措，让从严治党要求成为政府系统各级干部的“硬约束”。

　　进一步打牢宗旨观念，把人民满意作为第一标准，坚持问政于民、问需于民、问计于民，实现好、维护好、发展好人民群众的根本利益。

　　进一步做到求真务实，说实话、办实事、求实效，树立认真负责、敢于担当的干事态度，弘扬埋头苦干、勇于创新的工作精神，全面营造务实、诚信的政务环境。

　　进一步强化行政执行力，严肃抓落实的制度要求，完善抓落实的责任机制，坚决纠正有令不行、有禁不止行为，确保各项工作真正落到实处。

　　各位代表，新一轮振兴发展的大船已经扬帆起航。让我们在省委、省政府和市委的正确领导下，在市人大、市政协的大力支持下，紧紧依靠和团结全市人民，同舟共济，破浪前行，向着科学发展、加快振兴的宏伟目标昂首奋进！
